# Is There a Seamount Effect on Microbial Community Structure and Biomass? The Case Study of Seine and Sedlo Seamounts (Northeast Atlantic)

**DOI:** 10.1371/journal.pone.0029526

**Published:** 2012-01-18

**Authors:** Ana Mendonça, Javier Arístegui, Juan Carlos Vilas, Maria Fernanda Montero, Alicia Ojeda, Minerva Espino, Ana Martins

**Affiliations:** 1 Department of Oceanography and Fisheries, University of the Azores, Horta, Portugal,; 2 Instituto de Oceanografía y Cambio Global, Universidad de Las Palmas de Gran Canaria, Gran Canaria, Spain; 3 Instituto Canario de Ciencias Marinas, Gran Canaria, Spain; University of Hull, United Kingdom

## Abstract

Seamounts are considered to be “hotspots” of marine life but, their role in oceans primary productivity is still under discussion. We have studied the microbial community structure and biomass of the epipelagic zone (0–150 m) at two northeast Atlantic seamounts (Seine and Sedlo) and compared those with the surrounding ocean. Results from two cruises to Sedlo and three to Seine are presented. Main results show large temporal and spatial microbial community variability on both seamounts. Both Seine and Sedlo heterotrophic community (abundance and biomass) dominate during winter and summer months, representing 75% (Sedlo, July) to 86% (Seine, November) of the total plankton biomass. In Seine, during springtime the contribution to total plankton biomass is similar (47% autotrophic and 53% heterotrophic). Both seamounts present an autotrophic community structure dominated by small cells (nano and picophytoplankton). It is also during spring that a relatively important contribution (26%) of large cells to total autotrophic biomass is found. In some cases, a “seamount effect” is observed on Seine and Sedlo microbial community structure and biomass. In Seine this is only observed during spring through enhancement of large autotrophic cells at the summit and seamount stations. In Sedlo, and despite the observed low biomasses, some clear peaks of picoplankton at the summit or at stations within the seamount area are also observed during summer. Our results suggest that the dominance of heterotrophs is presumably related to the trapping effect of organic matter by seamounts. Nevertheless, the complex circulation around both seamounts with the presence of different sources of mesoscale variability (e.g. presence of meddies, intrusion of African upwelling water) may have contributed to the different patterns of distribution, abundances and also changes observed in the microbial community.

## Introduction

Seamounts are some of the most ubiquitous landforms on Earth and are present in uneven densities in all ocean basins [1]. Their peaks are found from a few up to thousands of meters below the surface. A recent study combining altimetry with the size-frequency relationship for larger seamounts estimates that there are about 125 000 (>1 km in height) seamounts across the globe [2], with only a few having been studied extensively so far.

**Figure 1:**
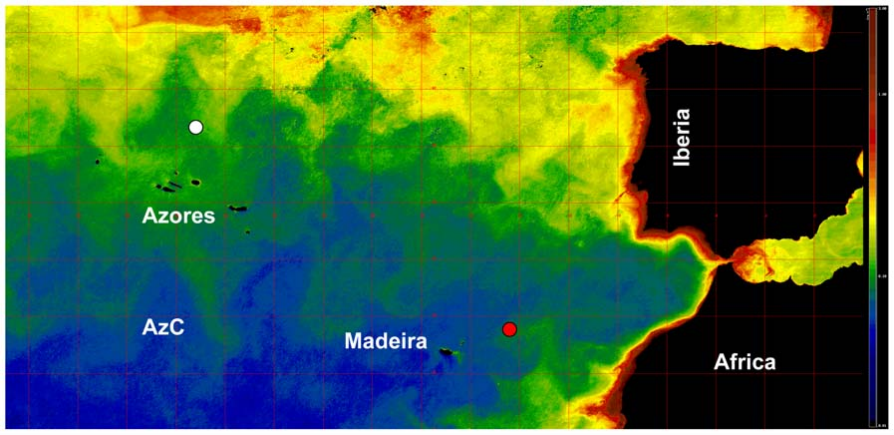
Monthly-averaged (July 2004) MODIS chlorophyll *a* (in mg m^−3^) image showing the geographical location of Sedlo (white circle) and Seine (red circle) seamounts. AzC: Azores Current.

Several decades of observational and modeling research have identified the distinct physical processes that occur at seamounts and have demonstrated the main physical forcing mechanisms behind these processes [3], [4]. These studies suggest that different seamount geometry, as well as the synoptic variability of impinging currents, result in a broad range of hydrodynamic patterns, relative strength and persistence of which may vary strongly in space and time [3]. As consequence, the integral effect of seamounts on biological communities becomes highly intermittent and difficult to access. The authors [5] studied 11 seamounts in the North and South Atlantic Ocean, concluding that each seamount was a unique case. For these reasons, the role of seamounts in oceans productivity is still not totally clear. Most seamounts occur in offshore, highly oligotrophic waters, away from continental influence and given this, enhancement of vertical fluxes, often associated with steep ocean bottom features, may lead to the injection of nutrients into the near-surface layer, vital to phytoplankton growth. The vertical uplifting of water over shallow seamounts can also increase the light levels experienced by phytoplankton, further increasing the possibility of locally enhanced primary production [6]. Comparing three seamounts located in the Pacific Ocean [7], the authors observed that the primary seamount effect on phytoplankton production and biomass appeared at the depth of the subsurface Deep Chlorophyll Maximum layer (DCM) with only occasional effects near surface. However, observational proofs of a persistent enhancement of primary productivity over seamounts have been lacking [8]–[12]. Our own research [12], carried out in the framework of the EU project OASIS (“Oceanic Seamounts: an Integrated Study”) showed a single sporadic increase in Chl a (and presumably in productivity) at Seine seamount (NE Atlantic) during a spring cruise, although during all the other cruises at Sedlo (NE Atlantic) and Seine seamounts (Fig. 1) the net community production was similar to values normally given for oligotrophic open oceans [13]–[16]. The same authors [12] suggested that closed circulation patterns over the top of the two seamounts should act preferentially as trapping mechanisms for organic matter, rather than being local sources of productivity.

Results from the OASIS project revealed also that both Seine and Sedlo seamounts offer highly complex hydrographical patterns [4], [17]–[18]. The two seamounts may receive upstream inputs of biologically important material or nutrients and have typical anti-cyclonic circulation around their summits, driven principally by Taylor-column formation, as tidal rectification is week at both seamounts [19]. The full observations at Sedlo by [4] showed that this circulation can be significantly disrupted by forcing of variable background flow and especially by the interaction of Meddies (i.e. anti-cyclonic eddies of warm, saline Mediterranean water”) impacting onto the seamount region. Monthly averages of satellite-derived Ocean Colour (OC) and Sea Surface Temperature (SST) for Sedlo and Seine regions during the period 1999–2006 [20] also show that both seamounts experience seasonal variation in temperature and chlorophyll a.

All these previous OASIS works provided detailed description of main physical forcing mechanisms affecting the two seamount environments, suggesting complex local hydrodynamics, and indicating the possibility of strong and variable biology shifts (namely in planktonic communities) in response to changing environments. In this paper we now analyze in detail the changes in microbial community structure and biomass, the contribution of each group to the whole autotrophic and heterotrophic biomasses, and their temporal and spatial variability on both seamounts. We aim to determine whether there are typical signatures of the microbial communities in each seamount and if microbial community abundance, biomass, or compositions are different with respect to the surrounding ocean.

## Materials and Methods

### Region of study and sampling strategy

**Figure 2:**
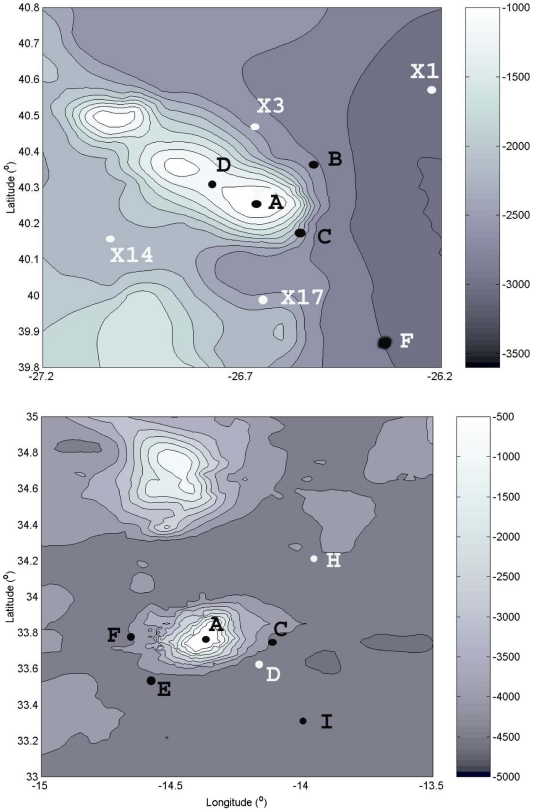
Bathymetry (m) of Sedlo (top) and Seine (down) seamounts showing stations positions. Stations F and X1 at Sedlo and stations H and I at Seine are considered reference “far field” stations.

Sedlo and Seine are both isolated oceanic seamounts in the Northeast Atlantic (Fig. 1) but differ in their geographic localization, topography, summit depths, and physical and hydrographic characteristics. Sedlo is a chain seamount composed of three summits, below the winter-mixed layer, with the shallowest at 760 m depth (Fig. 2). Seine is a cone-shaped seamount with a single summit at 175 m depth (Fig. 2), below the euphotic zone, but reaching into the winter-mixed layer [4]. Hydrographic characteristics at each seamount are described in detail by [4] and by [18]. Seine was sampled during November 2003, March and July 2004 (cruises: R.V. Meteor M60/1; R.V. Poseidon 309; and R.R.S. Discovery282, respectively); while Sedlo was sampled during November 2003 and July 2004 (cruises: R.V. Meteor M60/1 and R.R.S. Discovery282, respectively). During the sampling period of November 2003, a Meddy that collided with Sedlo in October 2003, was moving away to the southwest still influencing the seamount [4]. The sampling strategy consisted in a grid of stations centered at the seamount summit, extending to the flanks, and one or two reference far-field stations (Fig. 2). At each station water samples were collected at six different depths from surface down to 150 m depth, using Niskin bottles.

### Plankton cell counts

Microphytoplankton (>20 µm) and ciliates were observed and enumerated by inverted microscopy, at 400× magnification. Samples (125 ml) were fixed and preserved in a 1% final concentration of acidic Lugol solution, and settled in Uthermöl chambers for 48 h.

Seawater samples (30 ml) for autotrophic (NAF) and heterotrophic (NHF) nano-flagellates (2–20 µm) enumeration were preserved following [21]. Immediately after collection these samples were fixed with glutaraldehyde (0.3% final concentration). After 30 min, the samples were placed and filtered through a filtration system and fixed with proflavine (6.6 mg/ml final concentration) for 3–5 min. The stained sample was later filtered through a 0.2 µm black polycarbonate membrane filter, lying over a Whatman GF/C backing filter, and finally mounted on a microscope slide with low fluorescence paraffin oil. The slides were stored in dark in a −20°C freezer. Flagellates were counted using epifluorescence microscopy. NAF (palstidic) were distinguished from NHF (aplastidic) by their chloroplasts, which emitted red fluorescence when observed under blue light (excitation filter BP 450–490, chromatic divisor FT 510, suppressor filter LP 520). At least 50 cells or 20 fields were counted at a magnification of 1000×.

Heterotrophic bacteria (HB), small photosynthetic eukaryotic cells (picoeukaryotes, PE), and Prochlorococcus (Proc) and Synechococcus (Syn) type cyanobacteria, were counted by flow cytometry, using a FACScalibur (Becton and Dickinson) instrument, equipped with a 15 mW, 488-nm argon ion laser. Samples (4 ml) were fixed with 2% final concentration of paraformaldehyde, incubated for 15–30 min at 4°C and then stored frozen in liquid nitrogen until analyzed. To count HB, 200 µl was stained with a DMS-diluted SYTO-13 (Molecular Probes Inc.) stock (10∶1) at 2.5 µM final concentration. Bacteria were identified by their signatures in a plot of side scatter (SSC) versus green fluorescence (FL1). High DNA (H-DNA) bacteria and low DNA (L-DNA) bacteria were separated in the scatter plot as previously suggested by [22]. The identification of small phytoplankton groups (Proc, Syn and PE) was completed without stain addition. It was based on interactive analysis of multiple bivariate scatter plots of side scatter, red fluorescence and orange fluorescence. Samples were run at low speed for HB and at medium or high speed for phytoplankton, until 10,000 events were acquired. A suspension of yellow–green 1 µm latex beads (105 beads ml−1 for phytoplankton and 106 beads ml−1 for HB) was added as an internal standard (Polysciences, Inc.). Cells abundances were calculated from bead concentrations. The bead solution was checked daily through epifluorescence microscopy counting.

### Plankton conversion to biomass

The autotrophic biomass –expressed as particulate organic carbon (POC)- was calculated for the different plankton components. The community size structure fractions used were: POC_PE_ (picoeukaryotes), POC_Syn_ (*Synechococcus*), POC_Proc_ (*Prochlorococcus*), POC_HB_ (heterotrophic bacteria), POC_NAF_ (autotrophic nanoflagellates), POC_NHF_ (heterotrophic nanoflagellates), and POC_MICRO_ (the sum of diatoms, dinoflagellates and other microphytoplankton groups). The biomass of small heterotrophs was obtained by summing the POC_HB_ and POC_NHF_ biomasses. Large phytoplankton cells were converted to biomass from cell biovolumes, following [23]. The plasmar volume of diatoms was calculated according to [24]. The conversion to carbon was obtained multiplying the biovolume or plasmar volume by 0.11 according to [25]. Heterotrophic bacteria abundances were converted to biomass using a factor of 11.5 fgCcell^−1^ [26]. *Prochlorococcus* cell numbers were converted to biomass assuming a mean biovolume of 0.1 µm^3^ cell^−1^ [27], and a conversion factor of 290 fgC µm^−3^ [26]. *Synechococcus* cell numbers were converted to biomass by using a conversion factor of 100 fgCcell^−1^ [26]. The latter factor should be interpreted merely as an approximation since, as [27] observed, the conversion factor depends on the size of the cells, which increases with depth through the water column. Picoeukaryotes abundances were transformed to biomass using a conversion factor of 1500 fgCcell^−1^ [26]. Autotrophic and heterotrophic nanoflagellates numbers were converted to biomass assuming a mean biovolume of 14 µm^3^ cell^−1^ for both, and a conversion factor of 3080 fgC µm^−3^ [28].

The statistical significance of the difference in median biomass values for the different planktonic groups was tested. A two-sample *t* test was applied when the sampled populations had normal distributions and equal variances [29]. The non-parametric Mann-Whitney rank sum test was used as an alternative to a *t* test when the data were not normally distributed [29].

### Phytoplankton pigments and microplankton proteins

Chlorophyll a (Chla) and phaeo-pigments (Pha) were estimated fluorometrically according to [30]. Seawater samples (1 L) were filtered through Whatman GF/F filters. The filters' preservation and later analyses are described in [12]. Microplankton proteins (Pt) were determined according to the Peterson's modification [31] of the [32] method as also described in detail in [12].

### Microphytoplankton diversity

The microphytoplankton species diversity was estimated according to the Shannon-Wiener diversity index (H):
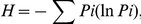
where Pi is the proportion of each species in the sample [33].

This index combines two quantifiable measures: the species richness (S) (i.e. the different species within the community), and species equitability – Evenness (E) (i.e. how even are the numbers of individual species) [34] where:
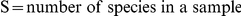


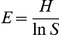



The significance in differences of the H index values was tested using the *t* test, following [34]. The *t* statistic associated with the H index is:
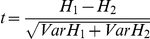
where H_1_ and H_2_ are the respective diversities of the two communities. Since the variance is an approximation, the *t* test should be referred to as an approximate test. The (adjusted) degrees of freedom (df) were calculated as:
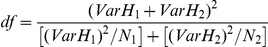



## Results

### Inter-seamounts variability

**Figure 3:**
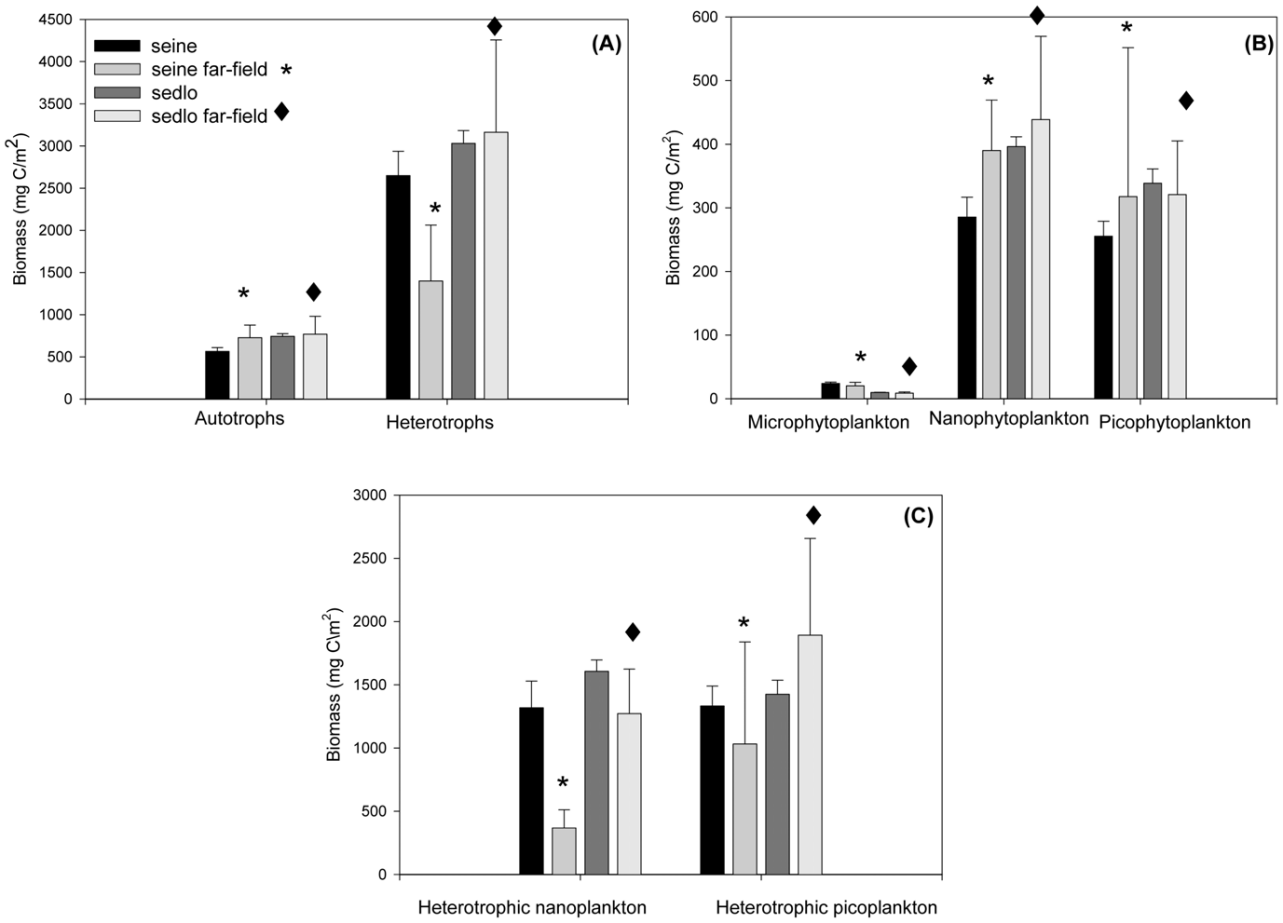
Biomass variability between seamounts and far-field stations. Water-column (0–150 m) integrated values (mg C m^−2^) averaged (±SE) from November 2003 and July 2004. (A) Total autotrophs *versus* total heterotrophs; (B) Autotrophs: microphytoplankton *versus* nanophytoplankton and picophytoplankton; (C) Heterotrophs: nanoplankton *versus* picoplankton.

For the same periods of sampling (November 2003 and July 2004), both seamounts show a clear dominance of the microbial heterotrophic community over the autotrophic one (H/A ratio >4), with a higher contribution in biomass of the smaller (<20 um) fractions (Fig. 3A). Nano and picophytoplankton clearly dominate over microphytoplankton, with a higher average biomass in Sedlo (Fig. 3B; Table S1). Conversely, microphytoplankton biomass is two fold higher in Seine. Like with autotrophs, the highest biomass of heterotrophs and the lowest variability are found in Sedlo (Fig. 3C). In all cases the “far-field” stations show always the highest variability. The Seine “far-field” autotrophic biomass is higher than stations within the seamount, but in Sedlo “far-field” and seamount stations have similar biomasses. In terms of heterotrophic community and during the common study period, the “far-field” stations have lower biomass than seamount stations in Seine but higher in Sedlo, (Fig. 3C).

### Seasonal variability

**Figure 4:**
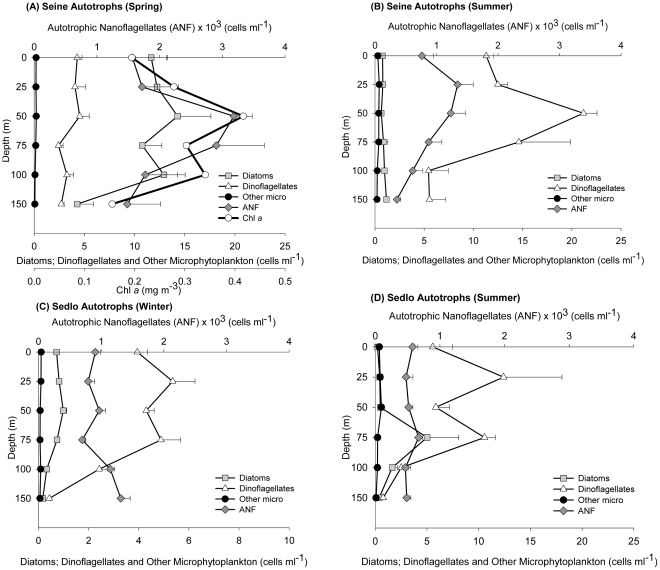
Vertical abundance distributions of micro and nanophytoplankton at Seine and Sedlo during different sampling periods. Values correspond to averaged data (±SE) from all stations around each seamount (excluding the far-field stations).

The seasonal vertical distributions in the average abundances of the different plankton size fractions for Seine and Sedlo are presented in Figs. 4 to [Fig pone-0029526-g005] 6. In general, higher abundances are found in the upper 75 m to 100 m layers, but the different microbial community groups show variable and complex distributions.

**Figure 5 pone-0029526-g005:**
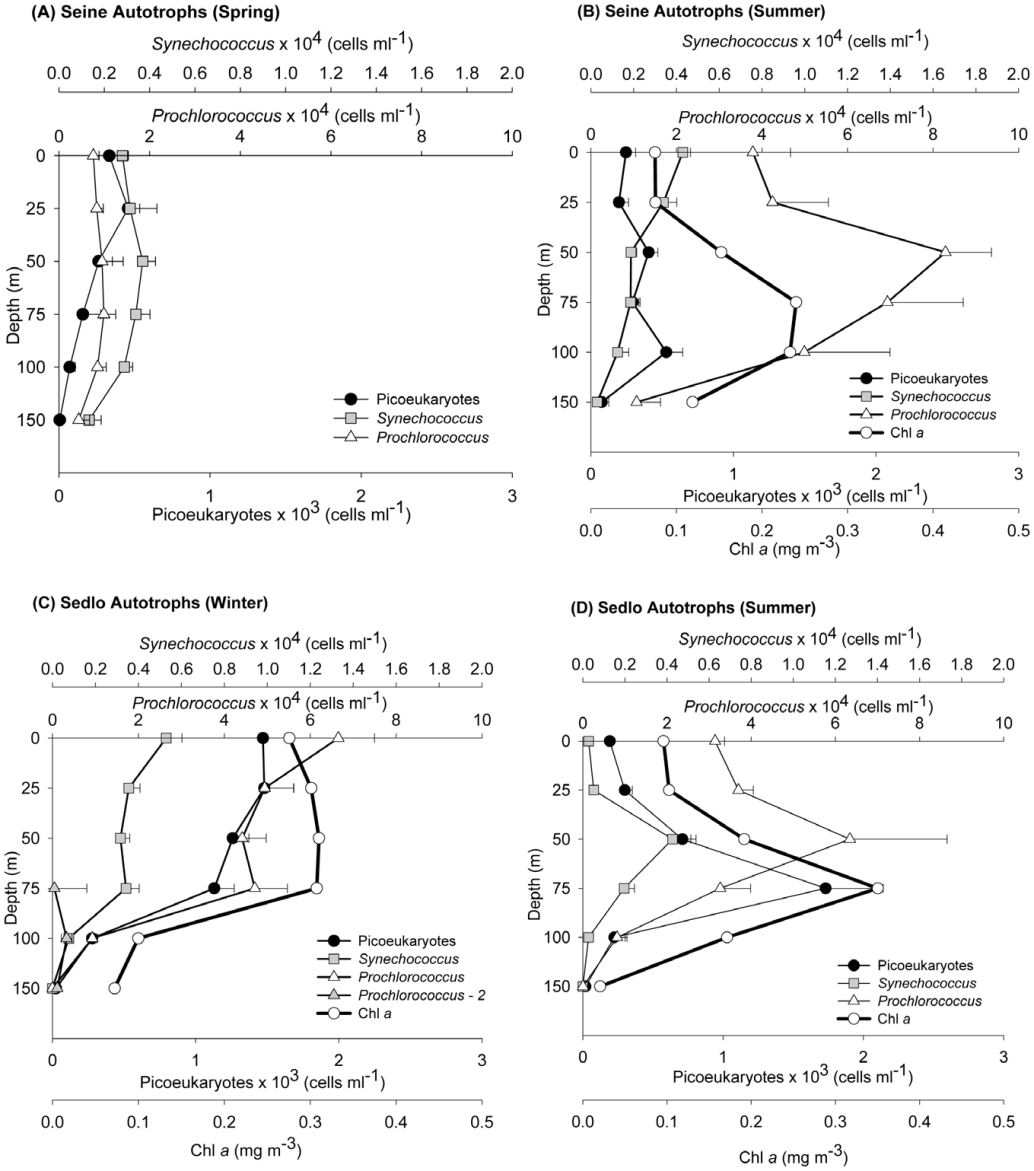
Vertical abundance distributions of eukaryotic and prokaryotic picophytoplankton at Seine and Sedlo during different sampling periods. Values correspond to averaged data (±SE) from all stations around each seamount (excluding the far-field stations). Vertical average profiles of Chlorophyll a (Chl a; mg m^−3^) are also added in B, C, and D.

**Figure 6:**
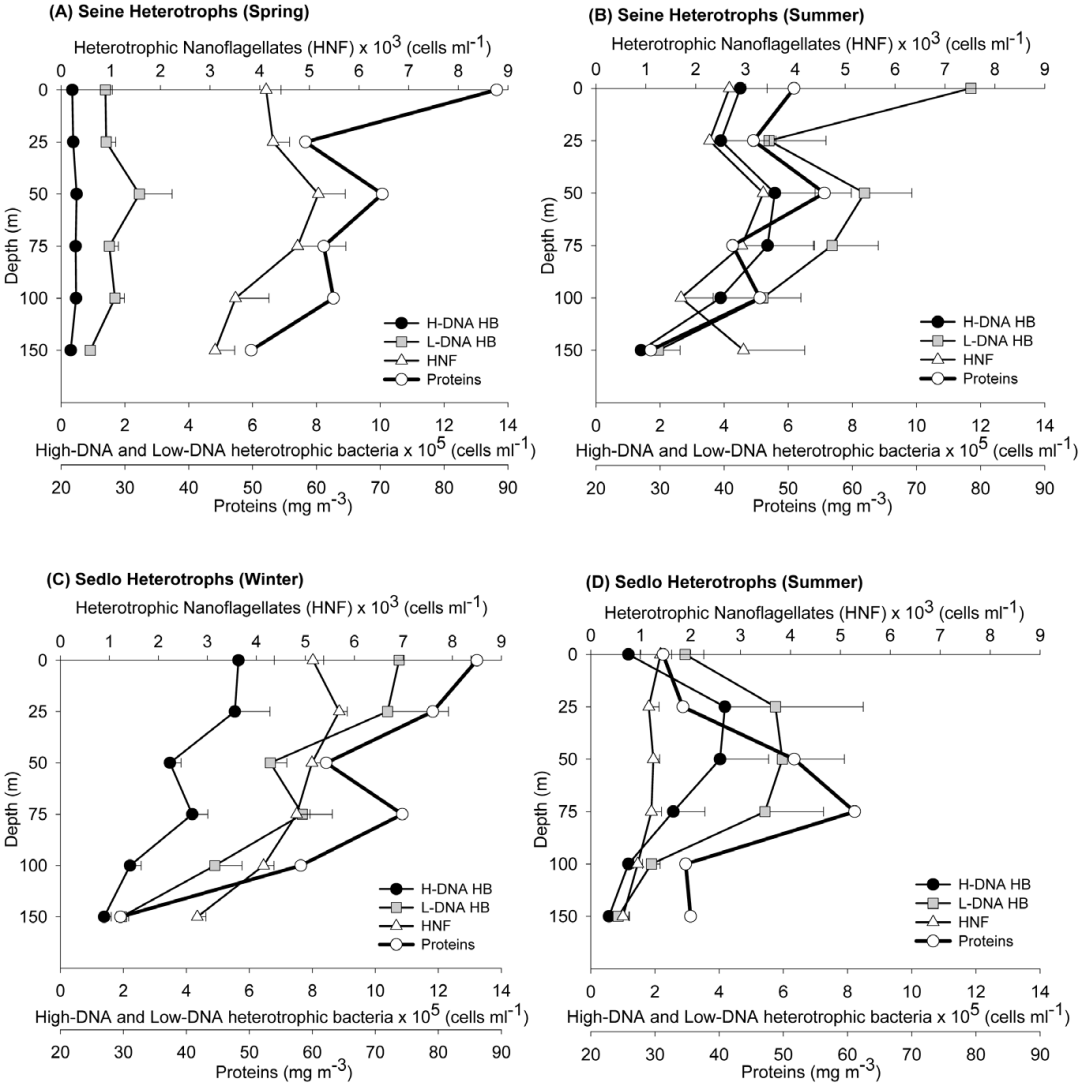
Vertical abundance distribution of heterotrophic planktonic organisms during different sampling periods. Values correspond to averaged data (±SE) from all stations around each seamount (excluding the far-field stations). Vertical average profiles of microplankton proteins (Pt; mg m^−3^) are also presented.

A phytoplankton increase is clear at Seine seamount during spring, with an increase in autotrophic community biomass of almost six fold the values found during winter (Table 1). This phytoplankton biomass is mainly composed by large forms (mainly diatoms and NAF), matching the vertical profile of Chla (Fig. 4A; Table S2). Both micro and nanophytoplankton present significant differences between March and November and March and July (Table S1). In fact, the microphytoplankton group has the highest relative contribution to total phytoplankton carbon (26%) observed in all periods (Table S3). During winter and summer, Seine autotrophic community biomass is dominated by picophytoplankton and nanophytoplankton, respectively (Table 1), with dinoflagellates dominating the microphytoplankton fraction (Table S2). Picoeukaryotes and *Synechococcus* show the highest integrated abundances during springtime at Seine (Fig. 5A and Table S2), while, in terms of abundance, *Prochlorococcus* dominate the picophytoplankton community during summer and winter (Fig. 5B–D and Table S2). A deeper population of *Prochlorococcus* (with larger cell size and higher fluorescence), named *Prochlorococcus-2*, was found on both seamounts only during winter (Fig. 5C and Table S2).

**Table 1:** Integrated (0–150 m) average (SE) biomass (mg C m^−2^) of the different plankton groups for Seine and Sedlo seamounts and far-fields, during November, March (only Seine) and July.

Plankton group	Month	Seine	Seine far-field	Sedlo	Sedlo far-field
*Prochlorococcus*	November	92 (17)	-	143 (14)	160 (6)
	March	35 (5)	29 (12)	-	-
	July	221 (30)	177 (40)	117 (18)	46 (2)
*Prochlorococcus-2*	November	17 (3)	-	3 (0)	16 (2)
*Synechococcus*	November	13 (1)	-	37 (4)	50 (6)
	March	43 (4)	26 (9)	-	-
	July	29 (3)	30 (14)	18 (2)	10
Picoeukaryotes	November	43 (6)	-	192 (16)	193 (17)
	March	418 (47)	214 (26)	-	-
	July	73 (6)	110 (64)	120 (16)	71
NAF	November	148 (31)	-	463 (15)	589 (13)
	March	980 (88)	453 (97)	-	-
	July	389 (45)	390 (40)	242 (16)	138
Diatoms	November	5 (1)	-	4 (0.2)	3 (0.3)
	March	488 (11)	380	-	-
	July	4 (0.2)	3 (0.3)	7 (1)	8
Dinoflagellates	November	26 (4)	-	2 (0.1)	2 (0.06)
	March	28 (5)	32	-	-
	July	12 (1)	17 (3)	4 (0.35)	5
Other microphytoplankton	November	3 (0.3)	-	1 (0.07)	1 (0.1)
	March	1 (0.08)	2	-	-
	July	1 (0.08)	0.5 (0.1)	2 (0.4)	0.6
H-DNA HB	November	252 (69)	-	552 (58)	743 (130)
	March	72 (4)	80 (15)	-	-
	July	722 (102)	393 (141)	394 (85)	144
L-DNA HB	November	386 (69)	-	1039 (101)	1911 (419)
	March	274 (43)	271 (38)	-	-
	July	1131 (132)	639 (262)	642 (141)	227
NHF	November	1543 (349)		2084 (43)	1677 (41)
	March	1898 (162)	1395 (208)	-	-
	July	1149 (388)	368 (72)	492 (27)	460

NAF: Autotrophic nanoflagellates; NHF: Heterotrophic nanoflagellates; H-DNA HB: High-DNA Heterotrophic bacteria; and L-DNA HB: Low-DNA Heterotrophic bacteria.

In Sedlo, nanophytoplankton and picophytoplankton represent the majority of the autotrophic biomass for all periods, with the highest biomass found in the NAF fraction during wintertime ( Table 1). Microphytoplankton is less represented, with small dinoflagellates dominating in abundance and large diatoms in biomass (Tables 2 and S2). The picophytoplankton match in general the Chl a profiles, with a local maximum at about 75 m depth, coinciding with the depth of the seasonal thermocline (Fig. 5C, D).

**Table 2:** Average plankton living biomass (B), total particulate organic carbon (POC), and contribution of B to POC at Sedlo and Seine seamounts.

Seamount	Biomass (B)	POC*	B/POC
	(mg C m^−2^)	(mg C m^−2^)	(%)
**Sedlo**			
November	4534	9828	46
July	2047	15060	14
**Seine**			
March	4238	23064	18
July	3748	22680	17

*POC: average value from 0–200 m [54].

The microbial biomass at both Seine and Sedlo is clearly dominated by the heterotrophic community during all seasons (Table 1). In Seine, a seasonal change in the heterotrophic community structure is observed from spring to summer, with a shift from high to low NHF and low to high HB (both L-DNA and H-DNA) abundances (Fig. 6A,B and Table S2). The main contribution to total heterotrophic biomass in spring and winter is from NHF, while HB dominates during summertime (Table S4). The pattern is different for Sedlo (Fig. 6 C, D). The highest averaged integrated abundance (Table S2) and biomass (Table 1) of the whole heterotrophic community is found during wintertime, with a significant increase of almost two and a half times in relation to summertime (Table S1). The heterotrophic vertical profiles show similarity with the proteins distribution, particularly with the HB profiles at Sedlo (winter) and Seine (summer) (Fig. 5A, D). L-DNA bacteria prevail over H-DNA populations at both seamounts and at all seasons sampled.

It is important to note that the seasonal variability is more marked at Sedlo far-field than at the seamount itself, with significant differences in all the components of the microbial community. On the contrary, the seasonal variability is similar at both, seamount and far-field stations, in Seine ( Table S1). Indeed, the global contribution of plankton biomass to POC_Total_ shows the highest and lowest percentages in Sedlo during winter and summer, respectively. In turn, Seine has very similar percentages among seasons (Table 2). Overall (i.e. for all cruises, seasons, and seamounts), the heterotrophic community represents the highest contribution to POC_Total_. One exception occurred in Seine during spring, when both communities contributed almost equally (9% in July and 10% in March).

### Intra-seamount variability

**Figure 7:**
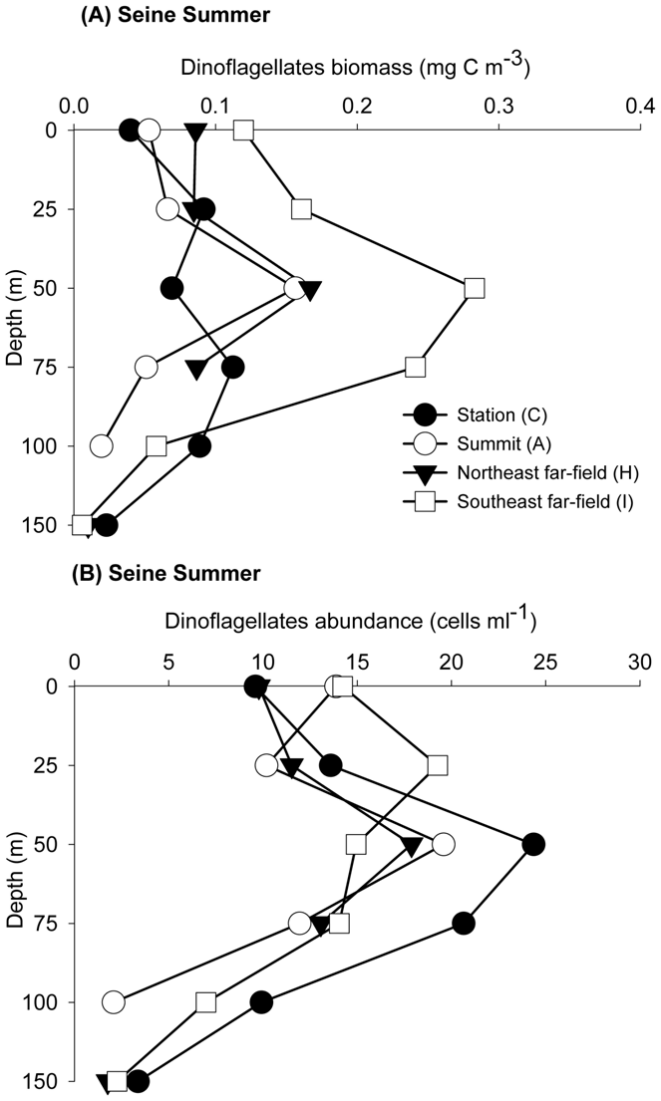
Intra-seamount variability in Seine. Variability in (A) dinoflagellates biomass (mg C m^−3^) and (B) abundance (cells ml^−1^) during July 2004. Comparisons are made between the summit station (“A”), a seamount station at the east margin (“C”) and the northeast and southeast far-field stations “H” and “I”, respectively.

Microbial community biomasses show large variability within seamount stations, and also between these and far-field stations (Table 1). In Seine, the maximum integrated autotrophic biomass is found at the seamount area in March, and this basically reflects the increase in large phytoplankton (Table 1). Autotrophic biomass is almost two times higher than at the far-field station (Tables 1 and S1). The overall microbial community shows also significant differences between Seine and the far-field stations (Table S1). The authors [12] found also a general increase of photosynthetic pigments and proteins at the summit of Seine (station “A”) in March. During July, autotrophic and heterotrophic groups present higher biomasses at stations near the seamount flanks or at the far-field station. As an example, the biomass of dinoflagellates is about three times higher at the far-field station “I” than at the seamount summit (Fig. 7A). Despite this, the highest integrated dinoflagellates abundance is not observed at station “I” but at station “C”, located in the east margin of Seine (Fig. 7B). During winter, the highest integrated biomasses of microphytoplankton, but lowest of picoeukariotes, were observed at the Seine summit station (not showed).

In Sedlo, plankton biomasses are in general larger at the seamount than at the far-field station during summer (Table 1), with significant differences for the overall microbial community (Table S1).Opposite to the summer season, the small picophytoplankton exhibit higher integrated biomasses at the far-field station “F” than within the seamount area in winter, (Table 1). Significant differences exist for almost all the microbial community groups between seamount stations and stations downstream and upstream the seamount (Table S1).

### Microphytoplankton diversity

The diversity (H) and evenness (E) from Sedlo and Seine seamounts are similar for November and July, the two common cruises (Table 3). No significant differences are found in H between the two cruises (Sedlo: t = 0,0049; df = 28344; and P>0,05; Seine: t = 0,7855; df = 3898; and P>0,05). Higher diversity and evenness is found during the spring cruise in Seine. In fact, significant differences are found in H between July and March (t = 16,5345; df = 68847; and P<0,05) and between November and March (t = 5,441; df = 3695; and P<0,05). For July and November cruises there are no significant differences in H between Sedlo and Seine (i.e. July: t = 1,5974; df = 35163; and P>0,05, and November: t = 0,0141; df = 5190; and P>0,05). The largest number of species is found in Seine during summer, due to dinoflagellates, but Sedlo is richer in species during winter.

**Table 3:** Shannon-Wiener Index (H), Species Richness (S) and Evenness (E) for microphytoplankton as a function of seamount and cruise.

	Seine	Sedlo
Shannon-Wiener Index (H)		
March	2.344	
November	2.175	2.176
July	2.150	2.176
Species Richness (S)		
March	103	
November	76	114
July	132	118
Evenness (E)		
March	0.506	
November	0.502	0.459
July	0.440	0.456

For all cruises and for both seamounts, intra-seamount microphytoplankton diversity shows significant differences between stations (not shown).

## Discussion

### Autotrophic vs. heterotrophic community variability

On both seamounts and during similar periods of sampling (summer and winter), our results show that the abundance and biomass of the heterotrophic community dominates over the autotrophic community. The authors [12] and [20] reported the presence of a phytoplankton bloom during March 2004 in Seine. This was the only circumstance in our study where the autotrophic community clearly increased, but even so, it did not exceed the heterotrophic community biomass (which contributed 53% to the total plankton biomass). These results support a previous study related with the plankton metabolic balance at Seine and Sedlo seamounts [12] where we found net heterotrophy most part of the time. Recent studies support the idea of predominance of heterotrophy in most oligotrophic regions [15], [35]–[39]. Based on published data [40], estimated a mean ratio of total heterotrophic to total autotrophic biomass (H∶A) for the open ocean of 1,85. In our study, the mean value for H∶A is two fold higher (3,79), but within the range given by the same authors (0,17 to 10,2). The authors [41] and [42] reported a three-fold lower value for H∶A for the NE Atlantic near Canary Islands region. In accordance with the results presented by [40] for the open ocean, our results also suggest an inverted biomass pyramid during most part of the time at both seamounts.

Seasonal shifts between NHF and HB contribution to total heterotrophic microbial biomass are well marked at the two seamounts, with a major contribution from HB during summer at Seine and Sedlo (62 and 68%, respectively), and minor (but still high) during winter at Sedlo (43%) and during spring at Seine (15%). The picoplankton heterotrophic community structure shows that L-DNA bacteria prevail over H-DNA populations at both seamounts and at all seasons sampled (Fig. 6 A–D and Table 1). There is some controversy regarding the activity of the H-DNA and L-DNA groups. The idea that H-DNA correspond to the actively growing fraction and L-DNA to the inactive fraction of a natural bacterial assemblage [41], [43]–[45] is not supported by results from Bouvier et al [46]. In fact, the last authors suggest a dynamic link between H-DNA and L-DNA bacteria fractions. In this work, 21% to 42% of the total bacteria abundance is from H-DNA bacteria. These values are consistent with other reported values for open ocean areas [47], [48].

In general, our results show higher (i.e. two to three-fold) bacteria abundance at both seamounts when compared with the results presented by [26] for the open Atlantic Ocean (between 30°N and 40°N). The authors [11] studied the microbial community in the Eastern region of the Subtropical North Atlantic (30°–34°N/27–31°W) nearby Seine, and reported higher integrated biomasses of H-DNA bacteria than ours. These authors used higher conversion factors than those used in this study (i.e. 20 fg C cell^−1^ versus 11,5 fg C cell^−1^, respectively), which may explain the main differences observed. Furthermore, our results show three times lower heterotrophic prokaryotes abundances in Seine during summer than those reported by [49] within a complex NW Africa-Canary Islands transition zone. The same authors attributed this increase to a strong frontal structure generated between an upwelling filament and the oceanic waters, probably due to the reported accumulation of particulate organic carbon at this front reported by [50].

In terms of autotrophic communities our results show that Sedlo is mainly characterized by nano and picophytoplankton, with a very small contribution of microphytoplankton to the autotrophic biomass. In Seine, small phytoplankton cells also dominate, but compared to Sedlo there was less pico and nanophytoplankton but more microphytoplankton. According to the picoplankton distributions defined by [26], Sedlo is located between typical northern temperate and northern Atlantic gyre provinces while Seine is located in the eastern margin of the northern Atlantic gyre province, relatively near the African coast. This means that the relative elevated contribution of large cells in Seine is most probably related to the influence of the African strong coastal upwelling that results in the thinning of the thermocline and concurrent intensification of coastal nutrient fluxes, thereby providing good conditions for large phytoplankton to develop. Furthermore, the high autotrophic and heterotrophic biomass variability found in Seine can be related to the highly dynamic hydrographical patterns in the region: i.e. the meandering of the Azores Current jet [4] and the influence of the Cape Guir filament in the African coast that propagates towards the ocean. The extent and influence of this upwelling event varies seasonally [51], as observed in ocean color remote sensing data. In particular, during the OASIS cruise in July 2004, a patchy filament was observed stretching the African coast towards the southern region of Seine seamount (Fig. 1). The filament is recurrently recorded during summertime, coinciding with the observed higher microbial biomass in Seine with respect to Sedlo. Conversely, during wintertime we observe higher microbial biomasses in Sedlo, suggesting the latitudinal effect reported by [52] for the Macaronesian islands and the remote sensing results obtained by [20] for these two seamounts (i.e. from more productive temperate waters in Sedlo to almost permanently stratified oligotrophic subtropical waters in Seine).

### Microbial community variability around Seine

Seasonal variability was observed in the biomass distribution of all plankton groups integrated over the first 150 m of the water column, with three times less picoplankton, two times more nanoplankton and 30 times more microphytoplankton in spring, with respect to summer. Particularly noticeable is the strong seasonal difference found in microphytoplankton biomass, being spring the period when this group attains the maximum contribution to total autotrophic biomass. The spring cruise was also the period characterized by the weakest vertical stratification, probably allowing the development of large autotrophic communities. A reduction was evident in the contribution to autotrophic biomass of large cells during winter and an increase of small cells in summer. A seasonal shift in microbial heterotrophic community was also evident at Seine from highest to lowest NHF and lowest to highest HB abundance and biomass from spring to summer.

A clear seamount effect in the microbial community was observed during spring, when all the autotrophic microbial community (with the exception of *Prochlorococcus*) was higher at the seamount stations than at the reference far-field ones. During this period, the microphytoplankton was mainly represented by diatoms, attaining the highest integrated biomass at the shallowest summit station. NAF biomass was three and two times higher than far-field stations H and I, respectively, during spring, but not in summer. Also picoeukaryotes increased about two times at seamount stations compared with far-field stations in spring, and about four times more than at the south far-field “I” station in summer. Nevertheless, in terms of spatial variability, no consistent results were found during the summer period.

We also observed increased heterotrophic biomass (HB and NHF) at seamount stations when compared with far-field stations, which probably contributed to increasing plankton metabolism. Indeed [12], reported high microbial respiration (Rd) rates in Seine during summer, and related this to organic matter loading from NW Africa upwelling system. Other authors [49], [53] also reported increases of heterotrophic prokaryotes and metabolism related with specific oceanographic features (e.g. strong frontal structures between coastal upwelling and offshore waters, or mesoscale eddies) when compared with surrounding waters.

Similar contribution of total plankton biomass to POC_Total_ is found at Seine, both during spring and summer (18 and 17%, respectively). The authors [54] already observed this lower contribution of total plankton biomass to POC _Total_ in Seine during spring, and related it to a higher proportion of detritus sinking material. In fact, these authors invoked the hypothesis of lateral advection of organic matter from a distant source, like the NW African upwelling region, supporting our own results.

### Microbial community variability around Sedlo

Summer and winter seasons exhibit distinct microbial community distributions. Contrary to Seine, the highest autotrophic biomass in Sedlo was found during winter (although no spring cruise was carried out in Sedlo). The principal contributors to POC_Chl_ were the NAF (with a biomass about two times higher than in summer) and PE. During wintertime, the picophytoplankton community increased in the upper 75 m depth, the lower limit of the winter seasonal thermocline. In general, during summertime the heterotrophic biomass almost tripled, with a shift from highest NHF (winter) to highest HB (summer) contribution to the whole heterotrophic community biomass.

The largest differences in the microbial distribution and biomass are observed between stations placed north and south of the seamount during wintertime. Pico, nano and microphytoplankton reveal higher abundances and biomasses on the southern side of Sedlo, while higher HB biomasses are found on the northern side. In particular, *Synechococcus* highest integrated biomasses are observed on the southern area of Sedlo, suggesting some sort of nutrient enrichment on surface waters. According to some authors [51], [55]–[57], *Synechococcus* is found at low concentrations in the oligotrophic subtropical oceans. However, intermediate abundances are observed in temperate and equatorial areas that transiently or permanently exhibit nutrient enrichment of surface waters, suggesting that it might be limited by low concentrations of inorganic nutrients. The authors [54] also observed significant differences in particulate organic matter between the northern and southern sectors of Sedlo during wintertime. According to [12], this variability could not be explained by any enhancement of local primary production. The authors [4] reported a meddy collision with Sedlo in October 2003. During our November cruise, this same meddy was moving away to the southwest still influencing the seamount [4]. This coincided with a marked positive vorticity, indicative of changing from anti-cyclonic (downwelling) to cyclonic (upwelling) circulation around the seamount summit. This change was related by [54] in the circulation pattern with the differential organic matter distribution observed at the northern and southern sectors of seamount. We believe this may explain the differences we also found between the north and south seamount microbial communities.

During summer, without the influence of a nearby meddy, a seamount effect is observed within the seamount area. This is reflected in the general increase in all microbial community groups biomass, when compared with the reference far-field station (i.e. “station F”). Peaks in biomasses of picoplankton (both phytoplankton and bacteria) were observed at the summit or at nearby seamount stations. The peak of *Prochlorococcus* at 50 m depth (station “A”, summit) coincides with an increase in water temperatures (i.e. between 17–21°C above 50 m depth) compared to the rest of stations. This agrees with the observation of [58] who reported that *Prochlorococcus* is most typical on oligotrophic regions of the oceans with water temperatures above 17°C.

The highest contribution of total plankton biomass (auto and heterotrophic community) to POC_Total_ is found during winter (46%) and the lowest during summer (14%). The authors [54] suggested that, contrary to Seine, advection of refractory carbon from allochotonous sources would be minor (if occurring) compared with vertical sedimentation of organic mater. This can probably explain the difference in the contribution of plankton biomass to POC_Total_ during summer and winter compared to Seine.

### Summary: Seamount effect

A clear seamount effect on microbial community structure and biomass was observed in both Seine and Sedlo seamounts under certain circumstances. In the first case, the effect is visible only during spring, with a local enhancement of large autotrophic cells, and the highest microphytoplankton diversity and evenness recorded at the summit and seamount stations. On the second case, and in spite of the existing low biomasses, Sedlo showed during summertime, clear peaks of picoplankton at the summit or at stations within the seamount area. Nevertheless, other superimposed factors such as mesoscale variability could also have contributed to the observed patterns of distribution, abundances and changes in community structure, masking any potential seamount effects. This is the case of Sedlo southern area, which was clearly influenced by the collision of a meddy during wintertime [4]. The same applies to Seine, where our results suggest the influence of the African upwelling system on the microbial community structure and biomass.

Compared to open ocean areas [40]–[42] both seamounts demonstrate a a clear two to three fold higher H∶A ratio.. Several authors [8], [9], [11], [54], [59], [60] report a trapping effect within a seamount area, which may be responsible for the accumulation of POM in the area. We believe that our results reflect this hypothesis and substantiate the important role of heterotrophic communities in oceanic seamount ecosystems. Nevertheless, improved sampling strategies are required to adequately resolve the different seamounts variability scales associated with oceanic areas, as each seamount is in fact, a unique case. Therefore, it is fundamental to support and maintain long-term multi-disciplinary oceanographic monitoring programs in these remote areas to fully understand their role in the surrounding ocean.

## Supporting Information

Table S1 Probability of significance (P<0.05*; P>0.05: not significant) of the difference in the averaged biomass values between paired comparisons of different planktonic groups and the overall plankton community.
(DOC)

Table S2 Integrated (0–150 m) average (SE) densities (cell cm^−2^) for the different plankton groups for Seine and Sedlo seamounts (excluding far-field stations) during November, March (only Seine) and July. NAF: Autotrophic nanoflagellates; NHF: Heterotrophic nanoflagellates; H-DNA HB: High-DNA Heterotrophic bacteria; L-DNA HB: Low-DNA Heterotrophic bacteria
(DOC)

Table S3 Contribution (%) of each autotrophic planktonic group to total autotrophic biomass.
(DOC)

Table S4 Contribution (%) of each heterotrophic planktonic group to total heterotrophic biomass.
(DOC)
