# Supplementary material for: Is There a Seamount Effect on Microbial Community Structure and Biomass? The Case Study of Seine and Sedlo Seamounts (Northeast Atlantic)
Source: PLoS One. 2012 Jan 18;7(1):e29526. doi: 10.1371/journal.pone.0029526 (PMC3261146; doi:10.1371/journal.pone.0029526)
Supplement: Table S1 — Probability of significance (P<0.05*; P>0.05: not significant) of the difference in the averaged biomass values between paired comparisons of different planktonic groups and the overall plankton community. (DOC) [file pone.0029526.s001.doc]

| **Variability** | **Seamount** | **Variable 1** | **Variable 2** | **Plankton groups** | **Significance** |
| --- | --- | --- | --- | --- | --- |
|  |  |  |  |  |  |
| Inter-SM | Seine+Sedlo | Seine | Sedlo | micro and nanophytoplankton | P>0.05 |
|  |  |  |  | picophytoplankton | P<0.05 * |
|  |  |  |  | nano and picoheterotrophs | P>0.05 |
|  |  |  |  | overall community | P>0.05 |
|  |  |  |  |  |  |
| Temporal | Sedlo | July | November | micro and nanophytoplankton | P>0.05 |
|  |  |  |  | picophytoplankton | P>0.05 |
|  |  |  |  | nano and picoheterotrophs | P<0.05 * |
|  |  |  |  | overall community | P>0.05 |
|  |  |  |  |  |  |
|  | Sedlo | July | November | micro and nanophytoplankton | P<0.05 * |
|  | (far-field) |  |  | picophytoplankton | P<0.05 * |
|  |  |  |  | nano and picoheterotrophs | P<0.05 * |
|  |  |  |  | overall community | P<0.05 * |
|  |  |  |  |  |  |
|  | Seine | March | November | micro and nanophytoplankton | P<0.05 * |
|  |  |  |  | picophytoplankton | P>0.05 |
|  |  |  |  | nano and picoheterotrophs | P>0.05 |
|  |  |  |  | overall community | P>0.05 |
|  |  |  |  |  |  |
|  | Seine | March | July | micro and nanophytoplankton | P<0.05 * |
|  |  |  |  | picophytoplankton | P>0.05 |
|  |  |  |  | nano and picoheterotrophs | P>0.05 |
|  |  |  |  | overall community | P>0.05 |
|  |  |  |  |  |  |
|  | Seine | March | July | micro and nanophytoplankton | P<0.05 * |
|  | (far-field) |  |  | picophytoplankton | P>0.05 |
|  |  |  |  | nano and picoheterotrophs | P>0.05 |
|  |  |  |  | overall community | P>0.05 |
|  |  |  |  |  |  |
| Intra-SM | Sedlo | seamount | far-field | micro and nanophytoplankton | P>0.05 |
|  | (July) |  | (F) | picophytoplankton | P>0.05 |
|  |  |  |  | nano and picoheterotrophs | P>0.05 |
|  |  |  |  | overall community | P<0.05 * |
|  |  |  |  |  |  |
|  | Sedlo | seamount | downstream | micro and nanophytoplankton | P<0.05 * |
|  | (November) |  | (X14, X17, F) | picophytoplankton | P<0.05 * |
|  |  |  |  | nano and picoheterotrophs | P<0.05 * |
|  |  |  |  | overall community | P<0.05 * |
|  |  |  |  |  |  |
|  | Sedlo | seamount | upstream | micro and nanophytoplankton | P<0.05 * |
|  | (November) |  | (X1, X3) | picophytoplankton | P>0.05 |
|  |  |  |  | nano and picoheterotrophs | P<0.05 * |
|  |  |  |  | overall community | P<0.05 * |
|  |  |  |  |  |  |
|  | Seine | seamount | far-field | micro and nanophytoplankton | P<0.05 * |
|  | (March) |  | (H, I) | picophytoplankton | P<0.05 * |
|  |  |  |  | nano and picoheterotrophs | P>0.05 |
|  |  |  |  | overall community | P<0.05 * |

Inter-SM: variability between seamounts; Temporal: variability between sampling periods; Intra-SM: spatial variability at a given seamount.
