# Supplementary material for: Is There a Seamount Effect on Microbial Community Structure and Biomass? The Case Study of Seine and Sedlo Seamounts (Northeast Atlantic)
Source: PLoS One. 2012 Jan 18;7(1):e29526. doi: 10.1371/journal.pone.0029526 (PMC3261146; doi:10.1371/journal.pone.0029526)
Supplement: Table S2 — Integrated (0–150 m) average (SE) densities (cell cm−2) for the different plankton groups for Seine and Sedlo seamounts (excluding far-field stations) during November, March (only Seine) and July. NAF: Autotrophic nanoflagellates; NHF: Heterotrophic nanoflagellates; H-DNA HB: High-DNA Heterotrophic bacteria; L-DNA HB: Low-DNA Heterotrophic bacteria (DOC) [file pone.0029526.s002.doc]

|  |  |  |  |
| --- | --- | --- | --- |
|  |  | **Seine** | **Sedlo** |
|  |  | **Average (SE)** | **Average (SE)** |
|  |  |  |  |
| *Prochlorococcus* (x10^4^) | November | 311 (94) | 494 (55) |
|  | March | 122 (28) |  |
|  | July | 721 (139) | 403 (93) |
| *Prochlorococcus-2* (x10^4^) | November | 54 (11) | 13 (2) |
| *Synechococcus* (x10^4^) | November | 13 (1) | 37 (5) |
|  | March | 43 (6) |  |
|  | July | 29 (3) | 18 (2) |
| Picoeukaryotes (x10^3^) | November | 29 (6) | 128 (12) |
|  | March | 279 (47) |  |
|  | July | 49 (5) | 80 (16) |
| NAF (x 10^3^) | November | 51 (13) | 141 (5) |
|  | March | 272 (23) |  |
|  | July | 126 (19) | 79 (8) |
| Diatoms | November | 20 (2) | 84 (8) |
|  | March | 1670 (303) |  |
|  | July | 133 (12) | 221 (91) |
| Dinoflagellates | November | 242 (28) | 477 (49) |
|  | March | 519 (70) |  |
|  | July | 1522 (124) | 830 (155) |
| Other microphytoplankton | November | 18 (2) | 11 (1) |
|  | March | 21 (5) |  |
|  | July | 44 (2) | 47 (2) |
| H-DNA HB (x 10^5^) | November | 219 (89) | 480 (59) |
|  | March | 62 (5) |  |
|  | July | 628 (118) | 342 (111) |
| L-DNA HB (x 10^5^) | November | 336 (90) | 904 (103) |
|  | March | 238 (56) |  |
|  | July | 885 (131) | 558 (184) |
| NHF (x 10^3^) | November | 535 (141) | 680 (15) |
|  | March | 616 (79) |  |
|  | July | 373 (168) | 160 (13) |
|  |  |  |  |
