# Supplementary material for: Is There a Seamount Effect on Microbial Community Structure and Biomass? The Case Study of Seine and Sedlo Seamounts (Northeast Atlantic)
Source: PLoS One. 2012 Jan 18;7(1):e29526. doi: 10.1371/journal.pone.0029526 (PMC3261146; doi:10.1371/journal.pone.0029526)
Supplement: Table S3 — Contribution (%) of each autotrophic planktonic group to total autotrophic biomass. (DOC) [file pone.0029526.s003.doc]

| **Seamount** | **POC_Micro_** | **POC_NAF_** | **POC_PE_** | **POC_Syn_** | **POC_Proc_** |
| --- | --- | --- | --- | --- | --- |
| **Sedlo** |  |  |  |  |  |
| November | 0.9 | 54.8 | 22.6 | 4.4 | 17.3 |
| July | 2.7 | 47.4 | 23.3 | 3.6 | 22.9 |
| **Seine** |  |  |  |  |  |
| March | 26.0 | 49.2 | 20.9 | 2.2 | 1.8 |
| November | 9.8 | 42.6 | 12.3 | 3.7 | 31.6 |
| July | 2.3 | 53.4 | 9.9 | 4.0 | 30.4 |

POC_MICRO_ (sum of the diatoms, dinoflagellates and other microphytoplankton groups); POC_NAF_ (autotrophic nanoflagellates); POC_PE_ (picoeukaryotes); POC_Syn_ (*Synechococcus*) and POC_Proc_ (*Prochlorococcus*).
