# Supplementary material for: Is There a Seamount Effect on Microbial Community Structure and Biomass? The Case Study of Seine and Sedlo Seamounts (Northeast Atlantic)
Source: PLoS One. 2012 Jan 18;7(1):e29526. doi: 10.1371/journal.pone.0029526 (PMC3261146; doi:10.1371/journal.pone.0029526)
Supplement: Table S4 — Contribution (%) of each heterotrophic planktonic group to total heterotrophic biomass. (DOC) [file pone.0029526.s004.doc]

| **Seamount** | **POC_HB_** | **POC_NHF_** |
| --- | --- | --- |
| **Sedlo** |  |  |
| November | 43.5 | 56.5 |
| July | 68.0 | 32.0 |
| **Seine** |  |  |
| March | 15.5 | 84.5 |
| November | 29.4 | 70.6 |
| July | 61.9 | 38.1 |

POC_HB_ (heterotrophic bacteria); POC_NHF_ (heterotrophic nanoflagellates)
